# Selections

**Published:** 1892-03

**Authors:** 


### Selections.

Painless and Unconscious Parturition.—Professor Tarnier (*Journal des Sages' Femmes*) describes a case where a woman came into his wards with a threatening shoulder-presentation. M. Chambreland rectified the malposition by external version, and brought down the head, though the membranes had broken half an hour before. A bandage, applied carefully around the body, kept the fœtus from returning to its malposition. A few hours later a child's cries were heard; the midwife in charge inspected the patient and, to the surprise of both, found the child, entirely delivered, between the thighs of the mother. Dr. Tarnier once delivered a woman who would persist in laughing and talking all the time ; the child was safely delivered after a few contractions, for they could not be called "pains," as she declared that they caused her no suffering. On another occasion he found a woman actually asleep in his ward, and the child, just born, lay between her legs. Another woman near term came to the hospital because she was alarmed at the rapidity of previous labors. He examined her, and found that labor was in progress. She expressed a desire to have an action of the bowels, but Dr. Tarnier forbade her to leave the room. She rose and tried to go out ; he held her back, and the child was suddenly delivered. The expulsion of the child was to the mother simply like passing a motion. Dr. Tarnier once opened a mammary abscess in a farm girl of herculean strength and proportions. He was asked to attend her during her confinement, and consented. But when sent for he found her delivered already. She declared that she felt no pain, simply something slipping away. He tried to extract the placenta, by traction, but she found the process ten times worse than the delivery of the child. Dr. Tarnier declares that a child can easily be delivered unconsciously into the pan of a water-closet. In such a case the cord will be torn across ; a clean cut would afford evidence of crime. The subject, however, is very difficult from a medico-legal aspect.—*Brit. Med. Jour.*

UPON THE MOST FAVORABLE TIME FOR FECUNDATION OF A WOMAN.—The time most favorable for fecundation is during the first day succeeding the menstrual period. The author bases this conclusion upon the examination of clinical cases, in which he was able to establish the precise date of the coitus which quite certainly resulted in fecundation. In six cases out of twenty-seven, coitus took place before the menstrual flow, but after confinement; it is probable that in five of these cases fecundation took place immediately after the menstrual flow; in the other twenty-one cases after confinement, it took place during the four days which followed. He next reports eleven cases of artificial fecundation after his process. Immediately after coitus, the woman still recumbent, he introduces a Cusco's speculum, the posterior valve of which is a little longer, so as to reach the posterior wall of the vagina. Of the spermatic fluid thus obtained by the valve, he places a third part in a syringe, like that of Braun, and injects it in the uterus. In all cases the injections were made before the menses, once only with success, which was on the day preceding the menstrual flow. In the other cases fecundation took place five times during the twenty-four hours that followed; once one day after; once two days after, and once three days after. The spermatozoids deposited in the vaginal cul-de-sac must have retained their vitality for seventeen days, and perhaps more. Their vitality is preserved very easily and for a very long time during the inter-menstrual period, but it can be proved also during the menstrual period.

We are, therefore, authorized to believe that in cases of pre-menstrual fecundation, there are spermatozoids remaining still alive since the close of the previous menstruation, which have passed into the uterus at a time appropriate for meeting the ovule. Moreover the limit of 300 days, fixed by law for legitimizing children in case of the absence or the death of the husband, must be below the reality, if account is to be taken of the time during which the spermatozoids remain inert, but still living, in the vagina before penetrating into the tubes and fecundating the ovule.—*Bossi Rivisti di ostetretica e ginecologia.—Weekly Med. Rev.*

WHEN TO STIMULATE.—The following are the physical heart signs which seem to indicate the early use of stimulants: 1. Early subsidence of the first sound, observed over the left ventricle. 2. Diminution of the first sound over the right ventricle. 3. The heart acting with a single, and that the second, sound. 4. Both sounds being audible, but their relative intensity being changed, so as to represent the action of the heart of a fœtus *in utero*. 5. With these signs, progressive diminution of impulse, which occasionally becomes imperceptible, even when the patient lies on the left side.—*Medical Age.*

---

HEREDITARY SYPHILIS.—The following was used by Mr. Jonathan Hutchinson with good effect in the case of an infant, some two months of age, afflicted with hereditary syphilis (*Archives of Surgery*, vol. ii., November 8, 1891):

℞.—Iodide of potassium................gr. ss.
Solution of bichloride of mercury.....m x.
Spirits of wine.....................m iv.
Water...........................ʒ j.
M. Sig.--To be taken three times a day.

The liquor hydrargyri perchloridi of the B. P. has one grain each of corrosive sublimate and chloride of ammonium, in two ounces of distilled water.

---

The *Dixie Doctor*, of Atlanta, we are informed, has aborted.—*Alabama Medical and Surgical Age.*

Mistake.—It did not abort, but by much labor and travail attained a speedy and premature menopause, and is now hopelessly languishing in the condition of innocuous menstrual desuetude. It now enjoys a calm and physiological functional repose which, in the course of nature, will doubtless be permanent.

---

DR. MORELL MACKENZIE, of London, England, died February 3, of pulmonary tuberculosis. Dr. Mackenzie's fame and skill were known throughout the world, and his special branch of medicine, diseases of the throat, knew no abler representative or more eminent authority. In 1888 he was knighted Sir Morell Mackenzie, as a reward for his faithful services to the Crown Prince of Germany. His diagnosis of the case secured to his illustrious patient the succession to the German throne. For this he was so bitterly censured that, upon the death of the Emperor, he prepared a history of his connection with the case, in some haste and excitement, under the title, the "Fatal Illness of Frederick the Noble."

